# Mutant Frequency is not Increased in Mice Orally Exposed to Sodium Dichromate

**DOI:** 10.14252/foodsafetyfscj.2018014

**Published:** 2019-03-13

**Authors:** Yasunobu Aoki, Michiyo Matsumoto, Michi Matsumoto, Kenichi Masumura, Takehiko Nohmi

**Affiliations:** 1 National Institute for Environmental Studies, Center for Health and Environmental Risk Research, Tsukuba, Japan; 2 National Institute of Health Sciences, Division of Genetics and Mutagenesis, Kawasaki, Japan

**Keywords:** genotoxicity, hexavalent chromium, *in vivo* mutagenesis, small intestine, transgenic rodent gene mutation assay, tumor

## Abstract

The *in vivo* mutagenicity of hexavalent chromium in the small intestine, the target organ of tumorgenicity, was examined by means of a transgenic mouse gene mutation assay. Sodium dichromate dihydrate was administered orally in drinking water to male *gpt* delta mice at a dose of 85.7 or 257.4 mg/L for 28 days or at a dose of 8.6, 28.6 or 85.7 mg/L for 90 days. No significant increase in *gpt* mutant frequency relative to that in control mice was observed in the small intestine in either the 28- or 90-day study, whereas 28-day oral administration of potassium bromate, a positive control substance, increased mutant frequency.

## Introduction

Hexavalent chromium compounds are categorized as Group I human carcinogens by WHO/IARC^1^^,^^2^^)^. Exposure to hexavalent chromium has been shown in epidemiological studies to increase the risk of lung cancer^3^^)^, while there is little evidence of an association between hexavalent chromium exposure and the incidence of cancer in gastrointestinal organs such as the stomach. Experimental animal studies conducted by the National Toxicology Program have shown that exposure to the hexavalent chromium compound sodium dichromate via drinking water for 2 years increases the incidence of tumors of the oral mucosa or tongue in rats and of the small intestine in mice^4^^)^. Therefore, the possibility of hexavalent chromium in drinking water to cause cancer in humans must be assessed.

Hexavalent chromium compounds are known to generate reactive oxygen species (ROS), which form oxidative adducts with DNA and proteins, resulting in activation of adverse outcome pathways such as genotoxicity and cytotoxicity^5^^)^. However, the mechanism and activating pathways contributing to the carcinogenicity of hexavalent chromium in rodents have not been studied. Hexavalent chromium compounds show mostly positive results both in Ames tests and in *in vitro* genotoxicity assays using cultured mammalian cells^6^^,^^7^^)^. In *in vivo* genotoxicity tests in rodents, hexavalent chromium compounds show negative results for micronucleus formation when administered via drinking water, whereas they show positive results in several *in vivo* tests after the gavage administration or intraperitoneal injection^6^^,^^7^^)^. Therefore, the *in vivo* mutagenicity of hexavalent chromium compounds in a target organ is necessary to be evaluated prior to assess the cancer risk posed by hexavalent chromium. In present study, we analyzed changes in mutant frequencies in *gpt* delta mice upon administration of sodium dichromate dihydrate via drinking water for 28 or 90 days, and observed no significant increase in mutant frequency relative to that in control mice in the small intestine, which is the target organ of tumorigenicity in mice.

## Materials and Methods

### Test Animals and Treatment Procedures

We purchased *gpt* delta mice, which carry approximately 80 copies of lambda EG10 on each chromosome 17 in a C57BL/6J background^8^^)^ (Japan SLC, Shizuoka, Japan). All animals were maintained under specific-pathogen-free and 12-h-light/12-h-dark conditions and received CA-1 chow (Japan Crea, Tokyo, Japan) *ad libitum*.

Sodium dichromate dihydrate (Na_2_Cr_2_O_7 _·2H_2_O, CAS No. 7789-12-0) (Nacalai tesque, Kyoto, Japan) was orally administered in drinking water to the *gpt* delta mice (male, 6 weeks old) at a dose of 0, 85.7, or 257.4 mg/L for 28 days, according to the procedure described in OECD Test Guideline 488 with slight modification^9^^)^, or at a dose of 0, 8.6, 28.6, or 85.7 mg/L for 90 days. The doses were selected based on the concentrations used in the 2-year cancer bioassay in mice^4^^)^. Four to six mice were used for each group. The sodium dichromate solution and the drinking water were changed every 3 or 4 days. During treatment, body weights and intakes of sodium dichromium solution and the water were measured. After the treatment, drinking water was provided *ad libitum* for 3 days to animals in the 28-day-treatment group and for 1 day to animals in the 90-day-treatment group, and then all the animals were euthanized.

Potassium bromate (KBrO_3_, CAS No. 7758-01-2) (Sigma-Aldrich, St. Louis, MO, USA), which induces tumors in the small intestine upon oral administration to mice^10^^,^^11^^)^, was used as a positive control. It was orally administered as drinking water to the *gpt* delta mice at a dose of 0 or 2 g/L for 28 days. After treatment, water was provided *ad libitum* for 3 days, and then the animals were euthanized. All animal care and handling procedures were conducted according to the Guideline for Animal Care and Use of the National Institute for Environmental Studies, and prior approval for all procedures was obtained from the Animal Care and Use Committee of the institute.

### Collection of Tissue

From each mouse, one-third (~10 cm) of the small intestine was excised from the stomach side, flushed with Dulbecco’s phosphate-buffered saline (PBS, Nissui, Tokyo, Japan), and cut for opening. After being gently rinsed with PBS to remove any intestinal contents and mucus, the mucosa was gently scraped from the intestinal wall. The collected mucosa was immediately frozen in liquid nitrogen and then kept at −80°C until the *gpt* mutation assay.

### *gpt* Mutation Assay

The *gpt* mutation assay was performed as described previously^12^^)^. Briefly, DNA was extracted from the small intestine mucosa by means of a RecoverEase DNA Isolation Kit (Agilent Technologies, Santa Clara, CA, USA), and lambda EG10 phages were recovered with Transpack Packaging Extract (Agilent Technologies). *Escherichia coli* YG6020 were infected with the recovered phages, plated on M9 salt plates containing chloramphenicol (Cm) and 6-thioguanine (6-TG), and then incubated for 72–90 h at 37°C. This incubation enabled selection of colonies harboring a plasmid carrying both the gene for chloramphenicol acetyltransferase and a mutated *gpt* gene. *gpt*-Mutant frequency was calculated by dividing the number of mutated colonies growing on agar plates containing Cm and 6-TG by the number of colonies growing on agar plates containing Cm alone. The mutants exhibiting the 6-TG-resistant phenotype were cultured overnight at 37°C in Luria–Bertani broth containing 25 µg/mL Cm, harvested by centrifugation (7000 rpm, 10 min), and then stored at −80°C. A 739-bp DNA fragment containing *gpt* was amplified by means of the polymerase chain reaction and sequenced as described previously^12^^,^^13^^)^.

### Statistical Analysis

All data are expressed as means with standard deviation (SD). Differences were examined by means of Student’s *t*-test; *P* < 0.05 was considered statistically significant.

## Results and Discussion

### Treatment for 28 days

**Fig. 1.**
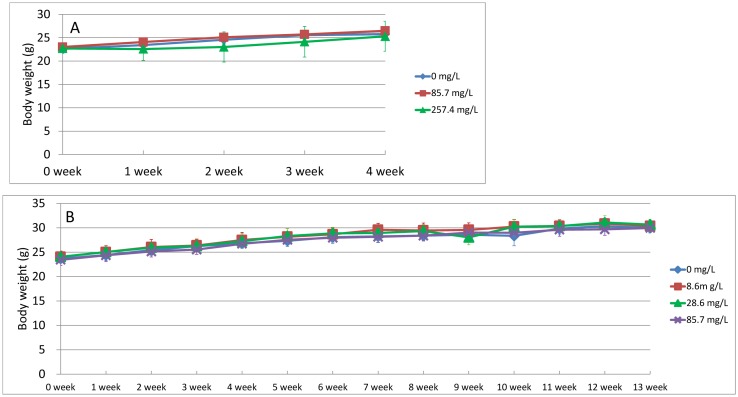
Changes in body weight of *gpt* delta mice during oral administration of sodium dichromate for (A) 28 days and (B) 90 days. Data are averages, and error bars indicate SDs.

To evaluate the mutagenicity of hexavalent chromium *in vivo*, sodium dichromate dihydrate in drinking water was given to *gpt* delta mice at a dose of 85.7 or 257.4 mg/L for 28 days according to OECD Test Guideline 488^9^^)^. These doses had been found to induce hyperplasia in the small intestine (duodenum) of male mice in a two-year cancer bioassay^4^^)^. During treatment, the body weight increase among the mice that received the 85.7 mg/L dose was similar to the increase among the control mice. The body weight increase of the mice that received the 257.4 mg/L dose was tend to be lower than that of the control mice, but did not differ statistically (Fig. 1A). The daily intakes of drinking water during the 28-day treatment period were estimated to be 12.6 ± 0.8, 10.5 ± 0.7, and 7.7 ± 0.6 mL for the control group, the 85.7 mg/L group, and the 257.4 mg/L group, respectively; These correspond to the average daily intake of sodium dichromate dihydrate is 0, 0.90, and 1.98 mg, respectively. The average daily intake of water for both of the treatment groups was significantly lower than that of the control group (*p* < 0.01).

Hexavalent chromium compounds had been known to induce tumor formation in the mouse small intestine^2^^,^^4^^)^. We thus expected that mutant frequency would be increased by oral administration of sodium dichromate at the tumorigenic dose in mice. After treatment with sodium dichromate for 28 days, no significant increase in mutant frequency was however observed (Table 1a); Average mutant frequencies were 0.58 ± 0.31 × 10^−5^, 0.96 ± 0.69 × 10^−5^, and 0.91 ± 0.45 × 10^−5^ for the control group, the 85.7 mg/L group, and the 257.4 mg/L group, respectively.

**Table 1a.** Mutant frequencies in the small intestine of *gpt* delta mice exposed to sodium dichromate via drinking water for 28 or 90 days.

Concentration	Exposure time (days)	Animal ID	Number of colonies		Mutant frequency (10^-5^)	Average mutant frequency ± SD (10^-5^)	
			Mutant	Total			
Control	28	1	5	603,200	0.83	0.58	±0.31
		2	2	544,640	0.37		
		3	7	823,650	0.85		
		4	3	1,158,000	0.26		
		**Total**	**17**	**3,129,490 **			
85.7 mg/L		1	11	596,000	1.85	0.96	±0.69
		2	2	701,800	0.28		
		3	4	1,182,000	0.34		
		4	5	336,300	1.49		
		5	12	1,436,030	0.84		
		**Total**	**34**	**4,252,130 **			
257.4 mg/L		1	16	1,672,650	0.96	0.91	±0.45
		2	8	480,850	1.66		
		3	5	947,650	0.53		
		4	8	1,048,460	0.76		
		5	5	796,500	0.63		
		**Total**	**42**	**4,946,110 **			
Control	90	1	6	549,933	1.09	0.80	±0.27
		2	9	1,960,000	0.46		
		3	10	1,455,000	0.69		
		4	8	1,017,750	0.79		
		5	23	1,991,633	1.15		
		6	7	1,135,000	0.62		
		**Total**	**63**	**8,109,316 **			
8.6 mg/L		1	9	1,335,000	0.67	0.62	±0.26
		2	12	1,707,750	0.70		
		3	7	822,467	0.85		
		4	5	1,945,000	0.26		
		**Total**	**33**	**5,810,217 **			
28.6 mg/L		1	11	1,810,533	0.61	0.49	±0.19
		2	10	1,758,000	0.57		
		3	8	1,406,167	0.57		
		4	4	1,900,000	0.21		
		**Total**	**33**	**6,874,700 **			
85.7 mg/L		1	9	1,262,800	0.71	0.77	±0.28
		2	18	2,250,000	0.80		
		3	3	270,000	1.11		
		4	7	1,595,000	0.44		
		**Total**	**37**	**5,377,800 **			

To confirm the insignificance of the mutant frequency between the control and treated groups, we estimated the mutation frequencies (the frequencies of independent mutant) after the treatment for 28 days by way of excluding the influence of clonal expansion of mutant in cell proliferation in the intestine. There was no significant difference in average mutation frequencies (the frequencies of independent mutation) between the control group and treated group as shown in Table 1b (0.58 ± 0.31 × 10^−5^, 0.74 ± 0.52 × 10^−5^, and 0.66 ± 0.34 × 10^−5^ for the control group, the 85.7 mg/L group, and the 257.4 mg/L group, respectively), indicating that no significant difference in mutant frequencies was not the influence of clonal expansion.

**Table 1b.** Mutation frequencies in the small intestine of *gpt* delta mice orally administered sodium dichromate for 28 days or 90 days.

Concentration	Exposure time (days)	ID of animals	Number of colonies		Mutation frequency (10^-5^)	Average mutation frequency ± SD (10^-5^)	
			Mutation	Total			
Control	28	1	5	603,200	0.83	0.58	±0.31
		2	2	544,640	0.37		
		3	7	823,650	0.85		
		4	3	1,158,000	0.26		
		**Total**	**17**	**3,129,490 **			
85.7mg/L		1	8	596,000	1.34	0.74	±0.52
		2	1	701,800	0.14		
		3	4	1,182,000	0.34		
		4	4	336,300	1.19		
		5	10	1,436,030	0.70		
		**Total**	**27**	**4,252,130 **			
257.4mg/L		1	10	1,672,650	0.60	0.66	±0.34
		2	6	480,850	1.25		
		3	5	947,650	0.53		
		4	6	1,048,460	0.57		
		5	3	796,500	0.38		
		**Total**	**30**	**4,946,110 **			
Control	90	1	6	549,933	1.09	0.66	±0.25
		2	9	1,960,000	0.46		
		3	10	1,455,000	0.69		
		4	7	1,017,750	0.69		
		5	13	1,991,633	0.65		
		6	4	1,135,000	0.35		
		**Total**	**49**	**8,109,316 **			
8.6mg/L		1	6	1,335,000	0.45	0.54	±0.25
		2	10	1,707,750	0.59		
		3	7	822,467	0.85		
		4	5	1,945,000	0.26		
		**Total**	**28**	**5,810,217 **			
28.6mg/L		1	10	1,810,533	0.55	0.46	±0.17
		2	10	1,758,000	0.57		
		3	7	1,406,167	0.50		
		4	4	1,900,000	0.21		
		**Total**	**31**	**6,874,700 **			
85.7mg/L		1	7	1,262,800	0.55	0.56	±0.13
		2	11	2,250,000	0.49		
		3	2	270,000	0.74		
		4	7	1,595,000	0.44		
		**Total**	**27**	**5,377,800 **			

Hexavalent chromium is a well-known ROS-generating agent, and thus possible to induce G-to-T transversion after treatment with sodium dichromate; This base substitution is associated with ROS via generation of 8-oxo-guanine^14^^)^. The results of our positive control study with potassium bromate showed that oral administration of this agent for 28 days significantly increased mutant frequency in the small intestine of *gpt* delta mice (Table 3; 0.35 ± 0.19 × 10^−5^ vs. 1.03 ± 0.53 × 10^−5^ for the control and treated groups, respectively; *p* < 0.05). This chemical induces small intestine tumors possibly through yielding oxidatively damages in DNA of DNA-repair-deficient mice and wild mice^10^^,^^11^^)^. Sequencing of the mutated *gpt* gene showed that G-to-T transversion was the major base substitution (41%) among the point mutations in the potassium-bromate-treated group, whereas G-to-A transition was the major mutation (46%) in the control group (Table 4). These results confirmed that oral administration of potassium bromate induced tumor formation in the mucosa of the small intestine^10^^,^^11^^)^ possibly through generation of ROS, as previously reported^15^^,^^16^^)^.

**Table 3.** Mutant frequencies in the small intestine of *gpt* delta mice exposed to potassium bromate via drinking water for 28 days.

Dose	Animal ID	Number of colonies		Mutant frequency (10^-5^)	Average mutant frequency ± SD (10^-5^)
		Mutant	Total		
Control	1	7	1,453,500	0.48	0.35 ± 0.19
	2	1	540,000	0.19	
	3	2	368,010	0.54	
	4	3	1,459,845	0.21	
	**Total**	**13**	**3,821,355 **		
2.0 g/L	1	12	628,650	1.91	1.03 ± 0.53*
	2	3	298,080	1.01	
	3	6	929,070	0.65	
	4	6	609,120	0.99	
	5	10	1,699,920	0.59	
	**Total**	**37**	**4,164,840 **		

*P < 0.05

**Table 4.** Spectrum of *gpt* mutations in the small intestine of *gpt* delta mice exposed to potassium bromate via drinking water for 28 days.

	Control		2.0 g/L	
Type of mutation in *gpt*	Number	%	Number	%
Base substitution				
Transition				
G:C → A:T	6	46	10	27
(CpG site)	(0)		(3)	
A:T → G:C	0	0	2	5
Transversion				
G:C → T:A	3	23	15	41
G:C → C:G	0	0	0	0
A:T → T:A	2	15	4	11
A:T → C:G	1	8	1	3
Deletion				
−1	1	8	5	14
≥2	0	0	0	0
Insertion	0	0	0	0
Other	0	0	0	0
*Total*	*13*	*100 *	*37*	*100 *

Administration of sodium dichromate for 28 days did not result in the increase in frequency of G-to-T transversion (24% and 17% for the 85.7 mg/L group and the 257.4 mg/L group, respectively) relative to the frequency in the control group (18%), whereas the frequencies of A-to-T transversion were higher in the 85.7 mg/L group and the 257.4 mg/L group (24% and 29%, respectively) than in the control group (18%), as shown in Table 2. The no apparent increase in the frequency of G-to-T transversion rather suggests that the ROS-generating activity of hexavalent chromium did not contribute to induce point mutations in the small intestine mucosa after oral administration for 28 days.

**Table 2.** Spectrum of *gpt* mutations in the small intestine of *gpt* delta mice exposed to sodium dichromate via drinking water for 28 or 90 days.

	Control		28 day								90 day									
	All (28 day + 90 day)		Control		85.7 mg/L		257.4 mg/L		All (85.7 + 257.4 mg/L)		Control		8.6 mg/L		28.6 mg/L		85.7 mg/L		All (8.6 + 28.6 + 85.7 mg/L)	
Type of mutation in *gpt*	Number	%	Number	%	Number	%	Number	%	Number	%	Number	%	Number	%	Number	%	Number	%	Number	%
Base substitution																				
Transition																				
G:C → A:T	34	43	6	35	12	35	10	24	22	29	28	44	14	42	9	27	13	35	36	35
(CpG site)	(23)		(3)		(8)		(5)		(13)		(20)		(9)		(4)		(4)		(17)	
A:T → G:C	4	5	0	0	2	6	6	14	8	11	4	6	2	6	0	0	1	3	3	3
Transversion																				
G:C → T:A	27	34	3	18	8	24	7	17	15	20	24	38	8	24	12	36	9	24	29	28
G:C → C:G	1	1	1	6	0	0	2	5	2	3	0	0	0	0	6	18	1	3	7	7
A:T → T:A	4	5	3	18	8	24	12	29	20	26	1	2	3	9	2	6	3	8	8	8
A:T → C:G	0	0	0	0	0	0	2	5	2	3	0	0	1	3	0	0	6	16	7	7
Deletion																				
−1	7	9	4	24	1	3	2	5	3	4	3	5	2	6	2	6	2	5	6	6
≥2	2	3	0	0	2	6	1	2	3	4	2	3	2	6	0	0	1	3	3	3
Insertion	1	1	0	0	1	3	0	0	1	1	1	2	0	0	2	6	1	3	3	3
Other	0	0	0	0	0	0	0	0	0	0	0	0	1	3	0	0	0	0	1	1
*Total*	*80*	*100 *	*17*	*100 *	*34*	*100 *	*42*	*100 *	*76*	*100 *	*63*	*100 *	*33*	*100 *	*33*	*100 *	*37*	*100 *	*103*	*100 *

‘All’ for control, 28-day, and 90-day indicates combined data of ‘control for 28-day study and 90-day study’, ‘85.7 mg/L group and 257.4 mg/L group in 28-day study’, and ‘8.5 mg/L group, 28.5 mg/L group, and 85.7 mg/L group in 90-day study’, respectively, and the columns of ‘All’ indicate the sum of number and the percentage of each mutation.

### Treatment for 90 days

Next, to determine whether mutant frequency was increased by longer-duration (subchronic) exposure, sodium dichromate was given to *gpt* delta mice via drinking water for 90 days. In this 90-day study, we used sodium dichromate doses of 8.6 mg/L as well as 28.6 and 85.7 mg/L (tumorigenic doses). The high dose in the 28-day study (257.4 mg/L) led to diminished increases in body weight and a decrease in daily water intake during treatment, suggesting that this dose induced systemic toxicity. During the 90-day treatment period, the body weight increases among the animals in the chromium-treated groups were similar to the increase in the control group (Fig. 1B). The daily intakes of drinking water during the 90-day treatment period were estimated to be 16.2 ± 0.9, 14.1 ± 1.3, 15.8 ± 1.2, and 15.2 ± 0.8 mL, of which the average daily intake of sodium dichromate dihydrate was estimated to be 0, 0.12, 0.45, and 1.30 mg, for the control group, the 8.6 mg/L group, the 28.6 mg/L group, and the 85.7 mg/L group, respectively; The average daily intake of water of the 8.6 mg/L group was significantly lower than that of the control group (*p* < 0.01).

Oral administration of sodium dichromate for 90 days did not increase the mutant frequencies in the groups treated with 8.6, 28.6, and 85.7 mg/L sodium dichromate (0.62 ± 0.26 × 10^−5^, 0.49 ± 0.19 × 10^−5^, and 0.77 ± 0.28 × 10^−5^, respectively) relative to the frequency in the control group (0.80 ± 0.27 × 10^−5^) (Table 1a), and the percentages of G-to-T transversion in the treatment groups (24%, 36%, and 24% for the 8.6 mg/L group, the 28.6 mg/L group, and the 85.7 mg/L group, respectively) did not differ significantly from the percentage in the control group (38%) (Table 2). After the treatment for 90 days, no significant difference was also observed in mutation frequencies between the control group and treated group as shown in Table 1b (0.66 ± 0.25 × 10^−5^, 0.54 ± 0.25 × 10^−5^, 0.46 ± 0.17 × 10^−5^, and 0.56 ± 0.13 × 10^−5^ for the control group, the 8.6 mg/L group, the 28.6 mg/L group, and the 85.7 mg/L group, respectively). The percentage of A-to-T transversion, which was higher in the treated groups than in the control group in the 28-day study, was not elevated in the 90-day study. These results indicate that a tumorigenic dose of hexavalent chromium did not increase the incidence of point mutations in the small intestine mucosa even when the exposure duration was prolonged to 90 days.

### Tumorigenicity of Hexavalent Chromium Independent of Its Mutagenicity

Hexavalent chromium compounds are categorized as human carcinogens, but their carcinogenic mechanism remains unclear. The genotoxicity of these compounds has been examined both *in vitro* and *in vivo*. Among Ames tests previously performed, almost tests show positive results, but results of some tests are negative, in the presence or absence of S9 mixture; and positive results have been observed in *in vitro* genotoxicity tests, such as chromosomal aberration tests and comet assay^4^^,^^6^^)^. Among *in vivo* genotoxicity tests, almost all micronucleus tests of a given hexavalent chromium compound show negative results in bone marrow cells and peripheral red blood cells upon exposure via drinking water, whereas hexavalent chromium compounds show positive results in comet assay when administered by gavage, as well as in chromosomal aberration, micronucleus, comet assay, and transgenic mice mutagenicity tests when administered intraperitoneally^6^^,^^7^^)^. That is, these tests give inconsistent results regarding the *in vivo* genotoxicity of hexavalent chromium compounds. Transgenic rodent gene mutation assays have remained to be tested in the target organs in the animals to which a hexavalent chromium compound was administered by drinking water.

In both of our studies (28- and 90-day exposures), oral administration of sodium dichromate did not significantly increase mutant frequency in the intestinal mucosa of *gpt* delta mice. Our results suggest that hexavalent chromium orally administered via drinking water is not mutagenic in the intestine, a tumor target organ in mice. Thompson et al. previously reported that oral administration of sodium dichromate to Big Blue^®^ transgenic rats for 28 days via drinking water did not significantly increase mutant frequency in the oral mucosa, a target organ in rats^17^^)^, or in the intestinal mucosa^18^^)^. Even after the treatment for 90 days via drinking water, K-Ras mutant frequency and micronucleus incidence did not increase in the mouse duodenum^19^^)^. These results indicate that hexavalent chromium compounds are not mutagenic in target organs, such as the small intestine, at the tumorigenic doses, and in turn suggest that the mutagenicity and related genotoxicity of hexavalent chromium compounds do not contribute to their tumorigenicity.

The tumorigenic mechanisms of hexavalent chromium have been investigated^5^^)^. If hexavalent chromium induces tumors by non-mutagenic mechanisms, ROS-generated cytotoxicity induced by these compounds may play a role in tumorigenesis. In fact, Thompson et al. observed a decrease in the reduced glutathione/oxidized glutathione ratio, as well as histopathological lesions, in the small intestine of mice upon oral administration of hexavalent chromium^20^^)^, and immunostaining of γ-H2AX (a biomarker of DNA damage) and chromium accumulation were increased not in the intestinal crypt compartment but in villus regions of mice^21^^)^. These findings suggest that oxidative stress, villous cytotoxicity, and crypt hyperplasia underlie the non-mutagenic mode of action for hexavalent chromium mediated intestinal tumorigenesis. However, further studies will be required to determine precisely whether the genotoxicity of hexavalent chromium contributes to the tumorigenic mechanism.
